# Improved Biogas Production from Human Excreta Using Chicken Feather Powder: A Sustainable Option to Eradicating Poverty

**DOI:** 10.1002/gch2.202100117

**Published:** 2022-04-11

**Authors:** Moses E. Emetere, L. Chikwendu, S. A. Afolalu

**Affiliations:** ^1^ Department of Mechanical Engineering Science University of Johannesburg Johannesburg 2006 South Africa; ^2^ Department of Physics Covenant University Canaan land Ota PMB 1023 Nigeria; ^3^ Department of Mechanical Engineering Afe Babalola University Ado Ekiti 360102 Nigeria

**Keywords:** biogas, enzymes, human excreta, microorganism

## Abstract

It has been proposed that providing energy for cooking and lighting would solve over 65% of energy needs in rural communities. The use of biomass resources has been found not sustainable as other bioproducts such as biodiesel and bioethanol depend on it. More so that there is a depletion of bioresources in some parts of the world. The shift into animal waste such as poultry droppings and cattle dung has huge prospects, but it is not sustainable in the long term as rural farmers depend on it. The use of human excreta is the most available and sustainable due to the human population. This research aims to provide a workable blueprint of biogas production to meet energy needs. The research considers a laboratory‐scale experiment whose result is used to project the medium‐scale biodigester. Microbial culturing from human waste is used to initiate the codigestion of human excreta and powdered chicken feathers. It is observed that this procedure drastically reduces the high nitrogen content in the biogas and improves its methane and carbon dioxide content. It is observed that the scaled‐up biodigester in a worst case scenario can function at 67%. Design parameters are documented for the onward adoption of the technique.

## Graphical abstract

This research proposes the easiest, cheapest, and sustainable route to produce biogas. Chicken feather is used to enhance the biogas quality and quantity from the anaerobic digestion of human excreta. The use of human excreta is found to be the most sustainable due to the human population.

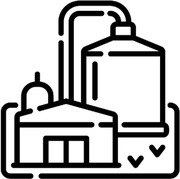

## 1 Introduction

Alternative energy is gaining the market's confidence as improved high‐performance energy trapping devices are sold. Among the top devices are solar photovoltaic (PV) modules and concentrators. However, the cost of such devices is comparatively high based on the poverty index of developing countries. 60% of the world's energy demand is required in developing countries whose current power generating capacity is unbelievable dismal. For example, the biggest hydropower station in Nigeria has a quoted capacity of about 7876 MW, but the working capacity is about 3000 MW or even less. As a result, the nation experiences massive load shedding.^[^ ^1^, ^2^ ^]^ The current status of power generation in Nigeria is attributed to inadequate power generation, delayed maintenance of facilities, obsolete equipment, inadequate equipment, lack of exploration, corruption, poor government funding, outdated grids, regular vandalism of the lines, lack of advanced technologies, inconsistency in billing, out of service transformers, and poor technical staff.^[^ ^3^ ^]^ Unfortunately, this scenario is replicated in many parts of the globe; hence, the immediate solution to the energy crisis globally is the empowerment of standalone users. Low purchasing and maintenance cost systems can empower standalone users in developing countries and the poor in developed countries. A self‐sustaining system is proposed, and its actualization was the focus of this research.

Much work has been done in biogas research. The main components of biogas that are generated from anaerobic digestion are methane and carbon dioxide (CO_2_). Biogas production is a four‐stage biochemical process comprising hydrolysis, acidogenesis, acetogenesis, and methanogenesis.^[^ ^4^ ^]^ U.S. Environmental Protection Agency (EPA)^[^ ^5^ ^]^ reported that food waste has three times the methane (CH_4_) production potential than most biomass with a yield (from anaerobic digestion) to be as high as 3200 standard cubic feet. Biogas generation from wastewater treatment plants (WWTPs) gained research interest with biogas yield potential of 450 MW or 2500 GWh per years.^[^ ^6^ ^]^ The codigestion of food waste, agricultural waste, or municipal waste and wastewater has shown huge biogas production and quality.^[^ ^7^, ^8^ ^]^

Attention is shifting to the human excreta as a sustainable biomass resource. Barman et al.^[^ ^9^ ^]^ characterized the human excreta and reported its components as fats (5–25%deciwatt(dw)), carbohydrates (10–30%dw), nitrogenous materials (2–3%dw), bacterial debris (10–30%dw), and inorganic matter (10–20%dw). Putnam (1971), in his work, characterized human urine and reported its components as inorganic salts (38%dw), urea (36%dw), organic compounds (13%dw), and organic ammonium salts (13%dw). The quality and quantity of biogas from human excreta will be determined by many parameters, including pH, temperature, feed composition, loading rate, mixing condition, reactor design, and residence time. One of the significant shortcomings of biogas from human waste is the high nitrogen content due to high ammonia concentrations.^[^ ^10^ ^]^ This challenge has been reported to affect the methanogens and syntrophic bacteria,^[^ ^11^ ^]^ which reduces the production of methane and carbon dioxide. This challenge is naturally resolved with time as the nitrogen is further oxidized to nitrogen oxides. The human excreta has a pH of 7.3, which is the optimum pH in biogas production.^[^ ^12^ ^]^ In other words, chemical treatment may not be an advisable solution as it will alter the PH of the substrate. Microbial treatment is suggested because it can be modified at various stages to suit whatever purpose. Microbes are microscopic organisms with four growth phases: adaptation phase, growth phase, stationary phase, and death phase. Fiery,^[^ ^13^ ^]^ clearly showed that the addition of rumen to substrates improves biogas production. The rumen is an inoculum that contains bacteria, fungi, archaea, and protozoa.^[^ ^14^ ^]^ However, it is not regarded as an effective microbe treatment because the high nitrogen content of the biogas persists.

In this research, we proposed a self‐sustaining system that would naturally optimize biogas production from human waste using cultured bacterial in human waste to biologically pretreat chicken feathers, increasing biogas production. Feather pretreatment can be done using several modern techniques, but the cost of such technology will be largely unfordable for low‐income earners across the globe. One of this research's objectives is to seek a solution that has zero cost implications for rural dwellers and low cost for urban dwellers. In this research, we proposed the use of powdered poultry feathers that can boost biogas yield and are affordable to low‐income earners.

The weight of chicken feathers is about 5–7% of the body weight.^[^ ^15^ ^]^ Dried feather contains 91% proteins and has 0.2 methane potential (m^3^ kg^−1^ VSadded) and 0.05 methane potential (m^3^ kg^−1^ wet weight).^[^ ^15^ ^]^ Chicken Feathers have a unique structure, and their special characteristics cannot be found in any natural or synthetic fibers. The feathers barbs have structures that make them suitable as a natural protein. The current applications of chicken feathers include their use in composites and nonwoven fabrics. It is used as an elegant piece to adorn clothes and accessories in the fashion industry. In the agricultural sector, feathers are used as animal feeds. They are blended and mixed for the massive production of feeds.^[^ ^16^ ^]^ Also, chicken feathers are used as fertilizers. They are used as fiber reinforcement for a poly (methyl methacrylate) matrix.^[^ ^17^ ^]^ It has been reported that chicken feathers are a good candidate for biogas production. This feat was achieved by altering the keratin structure for biogas production.^[^ ^18^ ^]^ The altering processes that have been used are chemical and biological pretreatment. Keratin has a high protein content that makes poultry feather an excellent raw material for biogas production.^[^ ^19^, ^20^, ^21^ ^]^

Sivakumar and Raveendran^[^ ^22^ ^]^ reported that bacteria such as *B. subtilis*, *B. licheniformis*, *E. coli*, *Klebsiella sp*., *S. aureus*, *Pseudomonas sp*., *Salmonella sp*., and fungi such as *A. flavus*, *A. fumigatus*, Trichophyton, and yeast could be used to degrade the keratin. Salminen et al.^[^ ^21^ ^]^ reported that 0.21 Nm^3^ kg^−1^ VS (volatile solids) could be obtained from untreated feather wastes. After treatment of the feather, several authors had reported that biogas production improved. For example, after biological pretreatment, Forgacs et al.^[^ ^22^ ^]^ reported a biogas yield of 0.31 Nm^3^ kg^−1^ VS (volatile solids).

## 2 Experimental Section

The material used for this study includes a syringe, plastic bottles, copper pipe of radius of 15 mm, adhesive gum, distilled water, solid human excreta, wooden cork, clock, thermometer, spring balance, and 250 mL bladder bag. These materials were selected as a prototype of the biodigester of the consumer. The experiment is a laboratory set‐up where basic parameters were adequately monitored for optimum performance.

**Figure 1:**
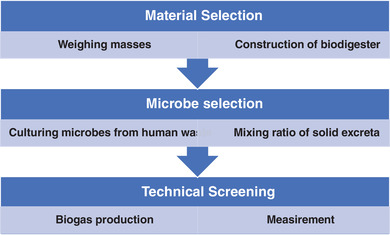
Flowchart of methodology.

The methods used for the study are illustrated in a flowchart below (**Figure** **1**). The steps include constructing laboratory‐scale biodigester, material screening, microbes screening, and technical screening.

**Figure 2:**
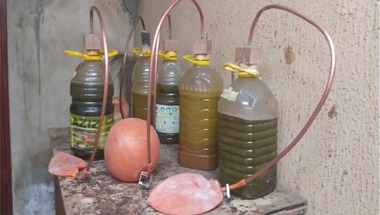
Laboratory set‐up of common biogas collection from human waste.

The laboratory‐scale biodigester was constructed, as shown in **Figure** **2**. The material in the construction was chosen to allow the steady flow of the gas produced to its endpoint. Five parameters were measured during this experiment, i.e., the ambient temperature and pressure in the laboratory, the mass of the biogas that is trapped within the balloon, the bacteria growth as seen in the increase of the digestate mass, and the time which was measured in twenty days (which excludes the preliminary days when no noticeable biogas generation was observed).

In this experiment, the chicken feather was used as the optimizing agent for improving the quality of biogas production from human excreta. The mixing ratio of the slurry of fresh human excreta was 1:5. The microbe was cultured for 15 days. There are trillions of microbes that reside in the human excreta.^[^ ^23^ ^]^ On day 15, it is expected that the microbes are at the stationary phase. 100 mL of the sludge was used for the experimental work.

The mixing ratio of the slurry of another batch of fresh human excreta was 1:5. Two different containers were labeled A & B. Containers A and B are made up of human excreta whose slurry gave a total of 4800 g. 100 mL of the cultured microbes were added to the slurry of both containers. 960 g of the powdered chicken feather was added to the slurry in container B, i.e., making the total mass as 5760 g. Hence, the ratio of chicken feathers to excreta was roughly 1:5. Each container was separately sealed, weighed, and connected to individual pipelines to allow the respective collection of gases. These containers are subsequently monitored for pressure building via opening the tap lock due to the nature of the container's material (plastic). The duration of this experiment spans 2 months. In the first 10 days of the experiment, no significant gas formation was recorded. The bladder remained flat throughout. In the 3rd week of the experiment, the bladder bag was half blown. In the 4th week, the bladder bag was fully blown. During this process, no external forces like shaking or heating were carried out in this experiment. The biogas was measured accordingly by weight and component.

Each gas sample produced from the experiment was analyzed using the RASI700 BIO Portable Gas Analyzer. Some readings are in ppm (part per million), while some are in %vol (percentage volume). The control experiment (pure human excreta) was measured using gas chromatography (GC‐MS), i.e., Finni‐gan Focus GC, ITQ 700, Thermo Electron Corp. The standard is highlighted in Knízek et al.^[^ ^24^ ^]^

The mixing ratio of the slurry can be calculated using Equation (1), while the biogas production can be calculated using Equation (2)^[^ ^25^ ^]^

(1) $$\begin{array}{c} V_{d}=\;\left(B+W\right)R_{t} \end{array}$$

*R*_t_ is the retention time in days, *V*_d_ is the volume of the bladder bag (mL), *B* is the biomass (kg), and *W* is the volume of water (mL)

(2) $$\begin{array}{c} G\;=G_{s}\;\times V_{f} \end{array}$$

*V*_f_ is the weight of feedstock, *G*_s_ is the gas yield, *G* is the biogas production. The assumed energy content of the produced biogas (E) as^[^ ^26^ ^]^

(3) $$\begin{array}{c} E\;=\;G\times \;0.006\frac{kWh}{dm^{3}} \end{array}$$

Or

(4) $$\begin{array}{c} E\;=\;G\times \;21.6\frac{kJ}{dm^{3}} \end{array}$$

## 3 Results and Discussion

The quality of the biogas production for human excreta (Sample A) was analyzed using the GC‐MS as shown in Table 2, which reveal that the flow rate of nitrogen, methane, carbon dioxide, propane, iso‐butane, and *N*‐pentane are 0.33, 0.045, 0.00 023, 3.75E‐9, 1.31E‐9, and 4.22E‐9 m^3^ s^−1^, respectively. Ethane, hydrogen sulfide, iso‐pentane, and *N*‐pentane had no flow rate hence no matter its amount in the gas, it may not flow into the bladder bag except its properties are altered. The average densities of nitrogen, methane, carbon dioxide, propane, iso‐butane, and *N*‐pentane are 1.33, 5.8, 66.3, 8008.4, 4961.1, and 1201.7 kg m^−3^. By their physical properties presented above, three gases would be more prominent in descending order, i.e., nitrogen, methane, and carbon dioxide. This categorization is not in any way related to the amount by percentage. Table 2 shows the inclusion of Iso‐Butane, *N*‐pentane, and Propane in the gas. These are usually called impurities or contaminations that somehow had contact with the gas. They are usually found in small quantities due to food consumed by the individual.

**Figure 3:**
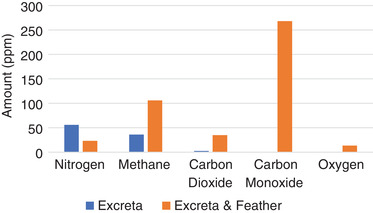
Comparative analysis of biogas content for excreta versus excreta/feather.

**Figure** **3** presents a chart that clearly shows the codigestion of human excreta and feather (HEF) versus human excreta only (HEO). It is observed that the addition of powdered feathers can reduce nitrogen content in the biogas by a minimum of 68%. This experimentation means that there could be minimum nitrogen content with more microbes in the human excreta acting on the chicken feather as biotreatment. Rajagopal et al.^[^ ^10^ ^]^ reported that nitrogen content inhibits anaerobic digestion at high ammonia concentrations. Scientists have reported that a high concentration of nitrogen ammonia is useful for bacterial growth, but it inhibits the growth of methanogenesis bacteria responsible for methane production.^[^ ^27^, ^28^ ^]^ This report is valid considering Figure 3 as the drastic reduction of nitrogen enhanced increases in methane production by 73%. The extensive effect of the microbes can be seen in the improved carbon dioxide and carbon monoxides content. It is observed that aside from the rich protein content of feathers, the trace metals are important ingredient that fosters the growth of methanogenesis bacteria. Gustavsson et al.^[^ ^29^ ^]^ and Qiang et al.^[^ ^30^ ^]^ reported that Fe, Co, and Ni enhance the growth of methanogenic bacteria. From **Table** **1**, it is clear how the trace metals in the feathers improved the biogas content.

**Table 1:** Element percentage in poultry feathers^[^ ^19^ ^]^

Element	Poultry liter	Feather meal	Feather hydrolysate
Ca	99.44	3146.48	8.73
Cu	4.09	32.64	0.12
Fe	333.44	576.23	0.45
K	11 616	25 520	250.0
Mg	1820	671.16	7.78
Mn	58.44	18.0	0.07
N	2110.67	119 484.76	1536.50

**Figure 4:**
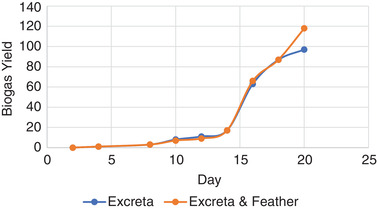
Experimental biogas production in HEF and HEO system.

The biogas production in both systems (i.e., HEF and HEO) is presented in **Figure** **4** below. It is observed that it took the microbes in human excreta about 18 days to pretreat the poultry feather. Hadiyarto et al.^[^ ^31^ ^]^ had reported that rumen microbes require up to 6–13 days to adapt to this substrate combination. The adaptation phase was influenced by inoculum size, microbial age, and environmental compatibility for microbial growth.^[^ ^32^ ^]^ In the case of microbes in human waste, it is unclear if the adaptation factors highlighted above are relevant. However, the microbes in human excreta are certainly dependent on the diet of the sources of the excreta. Microbes in human excreta that are salient for biogas production *etrasphaera, Trichococcus, Candidatus Microthrix, Rhodoferax, Rhodobacter, Hyphomicrobium, Methanobacteriales, Methanomicrobiales, Methanococcales, Methanocellales* and *Methanopyrales, Faecalibacterium prausnitzii, Roseburia intestinalis*, etc. The most important microbes in human excreta are methanogens. Guy et al.^[^ ^33^ ^]^ examined several faecal samples from healthy to unhealthy people. It was discovered that 5 out 8 persons produce methanogen. Sonja et al.^[^ ^34^ ^]^ reported that a total of 89% and 65% of adults and children, respectively, carried Methanobacteriales.

The implication of this result to small‐scale or domestic biogas production is the drastic reduction of biological treatment of chicken feathers. The microbes’ continuous production depends on the quantity of poultry feather powder introduced into the biodigester or sewage tank. After the microbes had significantly pretreated the feather, a clear distinction of the biogas production from HEF and HEO can be seen in Figure 4. Using the polynomial representation of biogas production in Figure 4, Equations (5) and (6) was generated as shown below

(5) $$\begin{array}{c} y\;=\frac{0.0001x^{6}+0.0091x^{5}+0.2576x^{4}-3.4512x^{3}+22.472x^{2}-64.844x+63.781}{225}\; \end{array}$$

(6) $$\begin{array}{c} y\;=\frac{4\;\times 10^{-5}x^{6}+0.009x^{5}+0.0141x^{4}-0.554x^{3}+5.23642x^{2}+17.858x+19.119}{225}\; \end{array}$$

where *x* is the number of days and *y* is the biogas production. Equation (5) represents the HEF system, while Equation (6) represents the HEO system.

**Figure 5:**
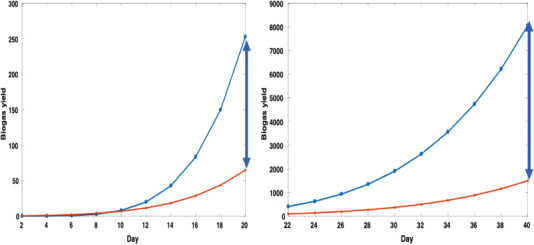
Theoretical biogas production in HEF and HEO system.

Theoretically, it is observed that the adaptation period for human microbes is 10 days (**Figure** **5**). This idea means this period will vary from one place to another, but the range may likely be 10–18 days. Against 120 g (or 0.48 g mL^−1^), a maximum of 250 g (or 1 g mL^−1^) can be achieved theoretically between 20 days. If the microbes continue to be active, it was observed that the biogas production within 40 days might be about 800 g (or 3.2 g mL^−1^). This result is moderate and significant to give biogas production range in a codigestive HEF system. This result is encouraging, i.e., compared to most codigestion systems in the literature. For example, the codigestion of donkeys, cattle, horses, and goats showed a maximum of 0.8 g mL^−1^ for 40 days.^[^ ^35^ ^]^

**Figure 6:**
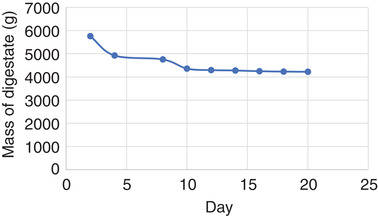
Digestate mass deduction.

The microbial population in the digestate was monitored by the mass variation presented in **Figure** **6**; It shows that there were weight losses which did not translate to the amount of biogas produced in the process. Also, digestate mass reduction may be ascribed to a depopulation of the microbes. This result is consistent with other studies that show a significant reduction in pathogens after anaerobic digestion.^[^ ^36^ ^]^

It was observed that there was a parabolic deduction of the digestate within the first 10 days of the anaerobic digestion. This result agrees with the theoretical prediction on the adaptation period of the microbes in humans to pretreat the chicken feather. This success may result from reducing the surface area of the feather by grinding it to powder. The mass of the digestate seems to converge toward 4200 g. 28% of the feedstocks are utilized for biogas production within twenty days and may likely increase to about 30% before it becomes redundant. The digestate is used for crop production; however, the interest of this research is centered on biogas production only. This calculation means that for every 10 000 tones of human excreta, there is likely 3000 tones of biogas produced about 18 MJ of energy on the laboratory scale.

## 4 Implication of Research on a Medium‐Scale Biogas Production Scheme

This study proposes a medium‐scale biogas production scheme for schools, farm settlements, clusters of houses, and religious centers in rural settlements. This project is in tandem with the Millennium Development Goal (MDG) number one, which bothers on eradication of extreme poverty, unemployment, hunger,^[^ ^37^ ^]^ and the Sustainable Development Goal number seven (SDG7) which bothers on clean energy. The vulnerability of low‐income urban dwellers in developing countries to intermittent power failure can indeed be attributed to poverty, unemployment, and corruption. The quest for standalone energy generation makes this project relevant to them. The dismal performance of energy generation programs in some developing countries has exacerbated its population's poverty rate because small and medium businesses are tied directly to energy. For example, the fruit seller needs energy for illumination at night, and the fish seller needs energy to preserve his/her goods, etc. Some literature ^[^ ^38^, ^39^ ^]^ reported that the average populace in some countries lives below $2 per day as the per capita energy consumption keeps deteriorating.^[^ ^40^, ^41^ ^]^ Salti and Chaaban,^[^ ^42^ ^]^ reported that the rate of poverty spread in some developing countries is alarming, resulting in 27% of its total population. In other words, the poverty index of a nation is directly proportional to its energy poverty which is defined as the lack of access to electricity and clean cooking facilities, which are two elements that are essential to meeting basic human needs.^[^ ^43^ ^]^

The formulation of Poverty Alleviation Programmes by notable organizations and government will be futility if the priority of basic energy provision is not first in its agenda. For example, the rural school system needs as much energy as schools in the urban center. The advancement in biogas production from feedstock such as human excreta and feathers would help to power automobile generators, thereby saving fossil fuel costs and polluting the environment. Also, the laboratories would be able to use the biogas for bunsen burners, heaters, light bulbs, cookers, laboratory equipment, and other needs. If the energy demand is met through this research, schools in rural settlements will be self‐sufficient as all feedstock are readily available within each locality. It is expected that this gesture will help the government or school proprietor to save about $400 monthly and about $5000 annually. Hence, this research is cogent and recommended for most countries globally as it is seen as the visible impact of waste‐to‐wealth.

Emetere et al.^[^ ^44^ ^]^ noted that the numbers of standalone energy users have significantly increased across the globe. In a way, this is a welcome development as energy management would be less cumbersome for energy providers, which are often overburdened by the high subscription of users. The only challenge would probably be the uncoordinated regulation of the energy sources of most standalone users. One of the initial indicators that enhanced the increase of standalone energy users in developing countries was inadequate planning on the side of the government. The photovoltaic technology was the first renewable energy source that standalone energy users in developing countries first embraced, but cost, environmental impact, and maintenance issues became a major shortcoming. Adopting biogas as an option for standalone users has better advantages because it is eco‐friendly and has a low maintenance cost. The advantage of this research is numerous but not limited to: huge feedstock resources that would not deplete over a lifetime; less maintenance of the biodigester or biodigester; saving money in the long run as the only cost that may be procured is the initial set‐up of the model; eco‐friendly of resulting bye products, i.e., it emits little or no waste products like greenhouse gases or pollutants into the air; aiding socio‐economic development, thus increasing investors’ confidence to investments. The sustainability of this research is predicated on three factors, i.e., the design of the biodigester, the continuous production of the microbes, and the feedstock ratio.

What do the results of this study portend to standalone users across the world? On a laboratory scale, for every 10 000 tones of human excreta used for this process, 6.25% would be generated in biogas while 93.75% would be digestate within 20 days. This percentage is expected to rise from 6.25% to 75% in the 32 days. The scenario is very different when the project is scaled‐up to a biodigester for medium‐scale businesses.

### 4.1 Design of Biodigester

**Figure 7:**
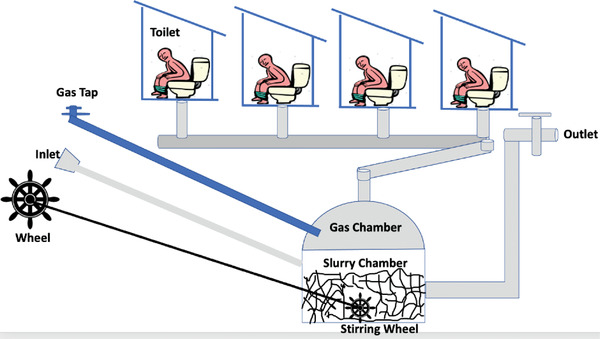
Model for medium‐scale biogas production scheme.

The model for this scheme is presented in **Figure** **7** below. Setting up this scheme is quite affordable as the only cost incurred is the wheel stirrer shown in Figure 7. Biogas digesters are mostly designed and constructed using bricks, cement, metals, and reinforced concrete, while in some cases, the dome of the gasholder is made up of fiberglass, reinforced plastic, and high‐density polyethylene (HDPE) plastic. In this model, the bricks are suggested for the slurry chamber, while the gas chamber is reinforced plastic. The digester chamber (slurry chamber) is cylindrical, and its volume is given as

(7) $$\begin{array}{c} V\;=\;\pi r^{2}h \end{array}$$

The gas chamber is dome shape and its volume is given as

(8) $$\begin{array}{c} V\;=\;\pi r^{2}h_{1}+\frac{1}{3}\pi r^{2}h \end{array}$$

The physics of this design is explained in existing literature.^[^ ^42^ ^]^ The construction of the biodigester is such that the main chamber is built as a septic tank for blocks of toilet. Hence, the feedstocks are added in discrete form, i.e., as the users use the toilet. The minimum requirement for the biodigester is presented in Table 3 below.

The wheel stirrer regulates the microbes, PH, and temperature of the feedstock at a certain interval. The ideal toilet system for this design should be air‐closet to reduce the water‐to‐solid ratio. However, the common toilet systems in urban and rural centers of developing countries are water closets and pit latrines. The water‐to‐solid ratio in pit latrines is moderate; however, there is the need to plan for the worst‐case scenario, i.e., water‐closet. In this case, the powdered feathers and the mechanical stirrer are two factors that make the design succinct for the objective of the project. The true biogas content in the water‐closet has appreciable water vapour content. Strömberg et al.^[^ ^45^ ^]^ postulated that water vapour increase biogas volume by 2–8% at normal temperature and pressure conditions. Edwiges et al.^[^ ^46^ ^]^ proposed a technique for estimating the normal volume of biogas at the normal condition as presented in Equation (9)

(9) $$\begin{array}{c} V_{o}=V\;\cdot \;\frac{\left(P_{L}-P_{W}\right)\;\cdot \;T_{0}}{P_{0}\;\cdot \;T} \end{array}$$

where *V*_0_ is biogas volume at normal conditions (mL); *V* is biogas volume recorded in the eudiometer (mL); *P*_L_ is atmospheric pressure at the time of registration (mbar); *P*_W_ is vapor pressure of water (mbar), and it is defined in Equation (10); *T*_0_ is the normal temperature (273 K); *P*_0_ is normal pressure (1013 mbar), and *T* is the temperature (K)

(10) $$\begin{array}{c} P_{w}=10^{8.962-\frac{1730.63}{T-39.724}}\; \end{array}$$

where *T* is the temperature (K). Abilene et al.^[^ ^47^ ^]^ made a derivation to determine biogas volume in a medium project using the Bernoulli equation, which is expected to augment the frictional loss term as presented in Equation (11)

(11) $$\begin{array}{c} V\;={\left(\frac{2gh(P+P_{w}-P_{0}}{K\rho }\right)}^{1/2}\; \end{array}$$

where *K* is an experimentally determined factor, which is the frictional loss factor of the gas valve, in this research, *K* was valued between 1 and 2. *K* = 1 occurs when the experiment is at its optimized state. However, at normal state, *K* = 1. “h” was the height of the gas chamber, and “g” was the acceleration due to gravity.

Moore and Holdeman^[^ ^48^ ^]^ reported that the bacterial component of the wet faecal mass is 30–40%. Alison and Cummin^[^ ^49^ ^]^ used the following methods (i.e., using electron microscopy, chemical analysis, microscopic counts, and light microscopy) to investigate the bacterial count. They reported that bacteria comprised of 54.7% of the total solids. This bacterial has the potential to exist for over 8 months.^[^ ^50^ ^]^

The relative bacterial activity factor can be calculated using the relation suggested by ref. [^51^], and it is given as

(12) $$\begin{array}{c} B\;=k_{4}\;\exp\left(k_{4}\times \left(T-k_{6}\right)\right)-k_{7}\exp\left(k_{8}\times \left(T-k_{6}\right)\right) \end{array}$$

where *B* is the relative bacterial activity factor (dimensionless); *T* is the operating temperature (°C); *k*_4_–*k*_8_ are model parameters that are obtained from Angelidaki et al.,^[^ ^51^ ^]^ where *k*_4_ = 0.96, *k*_6_ = 0.4, *k*_7_ = 5, and *k*_8_ = 0.26. Wu et al.^[^ ^52^ ^]^ gave the parameters as *k*_4_ = 0.494, *k*_6_ = 0.00 233, *k*_7_ = 0.323, and *k*_8_ = 23.8.

**Figure 8:**
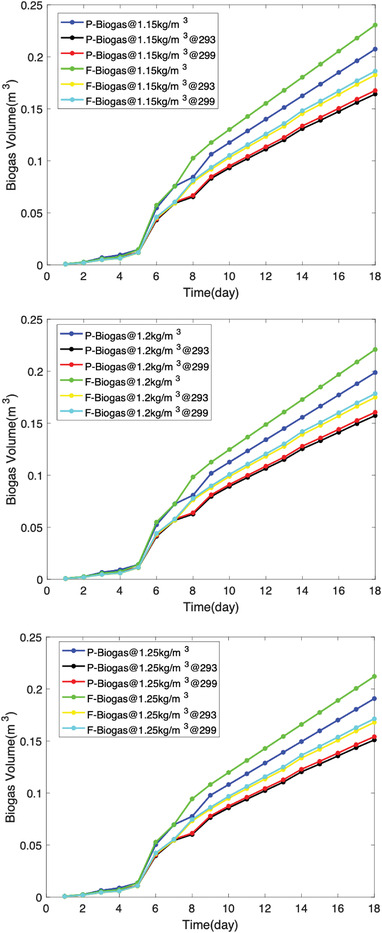
Laboratory biogas volume under varying biogas densities.

The biogas yield (in grams) was recalculated in the form of biogas volume using the biogas densities, which range between 1.15–1.25 kg m^−3^. The real biogas volumes were calculated using Equation (10), i.e., when the water vapor has been excluded under temperatures 293 and 299 K as presented in **Figure** **8**. Figure 8a presents a volumetric analysis for biogas from pure excreta (P‐Biogas) and codigested excreta (F‐biogas) at varying temperatures when the biogas density was 1.15 kg m^−3^. It is observed that temperature plays an important role in the anaerobic digestion of biogas from human waste. The higher the temperature, the higher the biogas yield expected, i.e., provided temperature does not exceed minimal temperature for the microbes. Second, it was observed that when the biogas density is about 1.15 kg m^−3^, the water vapor content in the biogas is <26%. This result is quite disturbing compared to the postulation of Strömberg et al.^[^ ^45^ ^]^ Hence, the mixing ratio of 1:5 in the laboratory may be inappropriate despite the addition of powdered feathers as feedstock. In a live scenario, i.e., in a water closet system, the powdered feather is expected to be much to improve the aqueous nature of the feedstocks. Compared to other biogas densities as presented in Figure 8a–c, it is observed that the lower the biogas density, the higher the volume of biogas expected. However, water vapor content is also expected to be lower in biogas of higher density. In a live scenario, the flow rate of the biogas component plays an important role in the collection of the biogas. For example, the volumetric flow rate has relevance to the flow rate shown in **Table** **2**.

**Table 2:** Default biogas analysis report

Component name	Time [min]	Height	Area [%]	Norm. area [%]	Raw amount	Amount [%]
Nitrogen	7.534	58 277.85	71.70	71.70	55.6110	59.09
Methane	8.299	23 820.43	26.06	26.06	35.9780	38.23
Carbon dioxide	9.605	1727.58	2.16	2.16	2.4724	2.63
Ethane	11.095	0.00	0.00	0.00	0.0000	0.00
Hydrogen sulfide	13.421	0.00	0.00	0.00	0.0000	0.00
Propane	15.171	9.49	0.01	0.01	0.0076	0.01
Iso‐Butane	19.885	14.58	0.03	0.03	0.0217	0.02
N‐Pentane	23.536	55.20	0.03	0.03	0.0199	0.02
Iso‐Pentane	35.115	0.00	0.00	0.00	0.0000	0.00
N‐Pentane	39.769	0.00	0.00	0.00	0.0000	0.00
		83 905.13	100.00	100.00	94.1105	100.00

The flow rate of nitrogen, methane, and carbon dioxide, which are 0.33, 0.045, and 0.00 023 m^3^ s^−1^, is expected to occupy the gas chamber in Figure 7. The calculations can be further calculated using the postulate given by Fedailaine et al.^[^ ^50^ ^]^ as presented in Equations (13)–(16)

(13) $$\begin{array}{c} K\;=Y_{m}\;\mu \chi \end{array}$$

(14) $$\begin{array}{c} \frac{d{\text{CO}}_{2}}{dt}=Y_{{\text{CO}}_{2}}\;\mu \chi \end{array}$$

(15) $$\begin{array}{c} \frac{d{\text{HO}}_{2}}{dt}=Y_{{\text{HO}}_{2}}\;\mu \chi \end{array}$$

(16) $$\begin{array}{c} \frac{dN_{2}}{dt}=Y_{N_{2}}\;\mu \chi \end{array}$$

where *Y*_m_ is methane production ratio (g/g), $Y_{{\text{CO}}_{2}}$ is carbon dioxide production ratio (g/g), $Y_{{\text{HO}}_{2}}$ is water vapor production ratio (g/g), and $Y_{N_{2}}$ is the nitrogen production ratio (g/g), μ is the rate of growth of anaerobic microorganisms (day^−1^), χ is the biomass concentration.

**Figure 9:**
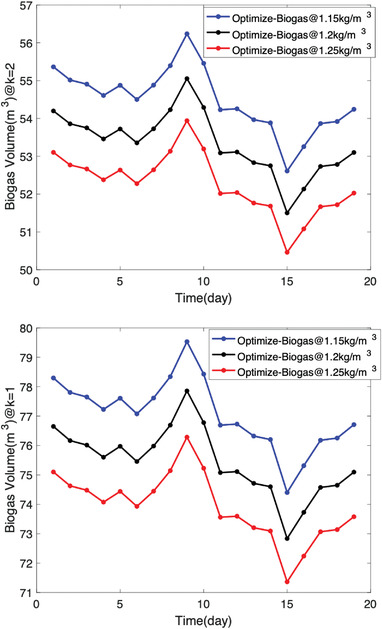
Model biogas volume under varying biogas densities.

**Figure** **9** is a presentation of live scenario as presented in the model, i.e., Figure 7. It is observed that the pressure gradient plays a vital role in the release of biogas during anaerobic digestion. When the experiment is at *K* = 1, it is an optimized state where it is assumed that the mixing ratio, bacterial activity, PH value, the temperature is at their normal state. In this case, it is expected that the biogas of density 1.15 kg m^−3^ will have a higher biogas yield than biogas densities of 1.2 and 1.25 kg m^−3^, which is about a 2% increase over each other. The significance of the 2% increase due to biogas density increase is evidence that the water vapour content estimated in the laboratory scale would be reduced by 2%. Hence, when expanded, the project has higher chances of success in meeting the energy demands of medium businesses in rural or urban centers.

**Figure 10:**
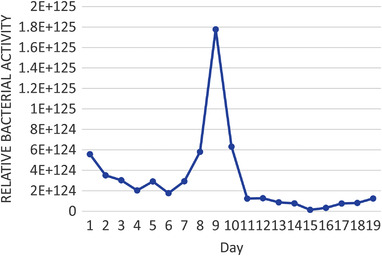
Diurnal relative bacterial activity.

In the normal situation where the operating conditions are dynamic, the experimentation assumes an experiment factor *K* = 2 as presented in Figure 9a. In this case, the biodigester operates at a reduced capacity of 41%. At this capacity, it is expected that the continuous mechanical stirring may improve the yield. Since it is most likely that the system operates at *K* = 2, Tables 4, 5, 6 illustrate the performance percentages in a live biodigester at different densities where the volumetric analysis of laboratory and biodigester scale is presented with emphasis on a biodigester whose volume is 89.3 m^3^ as shown in **Table** **3**. It was observed in **Tables** **4**, **5**, **6** that there are some days where the biogas yield from the codigestion process will yield lesser than normal. Within 19 days, it is expected that at least two days will have a drop in biogas yield. This result is largely because of the microbial activity and conditions in the biodigester under live operations. This scenario has been illustrated in **Figure** **10** where the diurnal relative bacteria activity is expected to be at its peak at a certain period and low at most times. In this regard, one way to flatten the curve is by mechanical stirring at intervals of some days. This action is expected to regulate the microbial activities within the biodigester.

**Table 3:** Specification of biodigester

Equipment	Radius [m]	*h*_1_ [m]	h [m]	The tensile stress of pipe [MPa]	Wall thickness of the pipe [mm]	mean diameter of the pipe [mm]
Specification	>2.5	≤2	4	52	≥2.2	≤110

**Table 4:**
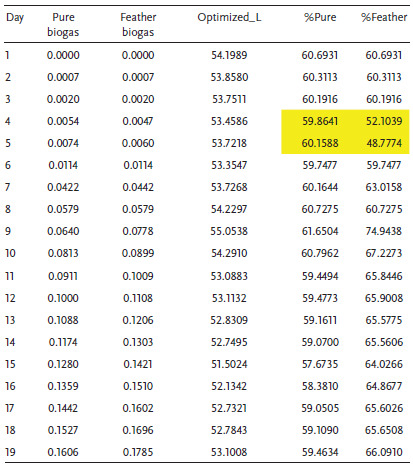
Biodigester performance at biogas density of 1.15 kg m^−3^

Considering Table 4, the average percentages of biogas yield of the P‐biogas and F‐biogas are 59.7% and 63%, respectively. The maximum percentages of biogas yield of the P‐biogas and F‐biogas are 61.7% and 74.9%, respectively. The percentages of biogas yield of the feather‐excreta codigestion at abnormal conditions is a good estimation for the actualization of the project as cost and maintenance is affordable in the long term. The minimum percentages of biogas yield of the P‐biogas and F‐biogas are 57.7% and 48.8%, respectively, which indicate that the diurnal relative bacteria activity is significant for the sustainable operation of the biodigester. Table 5 reveals that the average, minimum, and maximum percentages are the same as Table 4. In this case, the percentage of the daily biogas yield is almost the same for biogas density at 1.15 and 1.2 kg m^−3^. Considering Table 6, when the biogas density is 1.25 kg m^−3^, the average biogas yield of the P‐biogas and F‐biogas are 58.5% and 61%, respectively. The maximum P‐biogas and F‐biogas in percentages are 60.4% and 73.4%, respectively. The minimum biogas yield of the P‐biogas and F‐biogas is 56.5% and 47.8%, respectively. The biogas at density 1.25 kg m^−3^ is observed to have lower percentages of biogas yield than lower biogas densities. However, the lower magnitude of standard deviation is somewhat advantageous for biogas at 1.25 kg m^−3^.

**Table 5:**
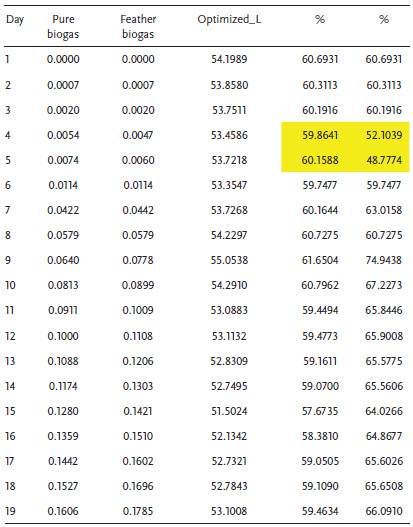
Biodigester performance at biogas density of 1.2 kg m^−3^

**Table 6:**
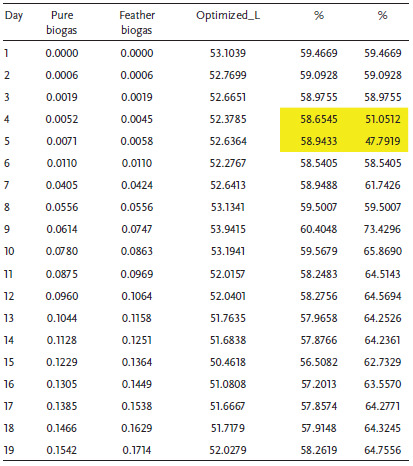
Biodigester performance at biogas density of 1.25 kg m^−3^

The diurnal relative bacterial activity trend for the biodigester is somewhat generic because of the stages in microbial growth. This experiment further confirmed that one of the ways to flatten the peak of the diurnal relative bacterial activity is to culture the adaptation period externally before introducing it into the biodigester. This process is envisaged to extend the growth stage within the biodigester to improve the daily biogas yield.

## 5 Conclusion

The driving objectives of low cost and enhanced biodigester were achieved by optimizing human biogas from human waste using agro‐based products (feather). The progression of a laboratory scale to a medium reactor scale for medium businesses in rural and urban centers of developing countries has been proven achievable with a daily yield as high as 69%. Salient observations were made. The laboratory‐scale observed that individual gaseous components in the biogas had a flow rate of 0.33, 0.045, and 0.00 023 m^3^ s^−1^ for nitrogen methane carbon dioxide. Also, it was seen that there was the presence of impurities or contaminations in the biogas, which is likely due to the diet of the individual whose excreta was used for this work. The microbial activities drastically reduced nitrogen content in the biogas and enhance methane production by 73%. The extensive effect of the microbes can also be seen in the improved carbon dioxide and carbon monoxides content. In the biodigester scale, it was observed that the higher the temperature within the biodigester, the higher the biogas yield expected, i.e., provided the temperature does not exceed minimal temperature for the microbes. Also, it was observed that when the biogas density is about 1.15 kg m^−3^, the water vapour content in the biogas is <26% and is reduced when there is conscious feed stocking of poultry feathers and mechanical stirring of the feedstock. The specification and operations of the biodigester are recommended for the pilot project across medium businesses in rural and urban centers in developing countries.

## Conflict of Interest

The authors declare no conflict of interest.

## Data Availability

The data that support the findings of this study are available from the corresponding author upon reasonable request.
